# Evaluation of two rapid molecular test systems to establish an algorithm for fast identification of bacterial pathogens from positive blood cultures

**DOI:** 10.1007/s10096-020-03828-5

**Published:** 2020-02-04

**Authors:** Philipp Oberhettinger, Jan Zieger, Ingo Autenrieth, Matthias Marschal, Silke Peter

**Affiliations:** 1 Institute of Medical Microbiology and Hygiene, University of Tübingen, Elfriede-Aulhorn-Str. 6, 72076 Tübingen, Germany; 2 German Center for Infection Research (DZIF), Partner Site Tübingen, Tübingen, Germany

**Keywords:** Bloodstream infection, Molecular identification, Antibiotic resistance, Rapid identification system, GenMark ePlex®, Biofire FilmArray®

## Abstract

Fast identification of pathogens directly from positive blood cultures is of highest importance to supply an adequate therapy of bloodstream infections (BSI). There are several platforms providing molecular-based identification, detection of antimicrobial resistance genes, or even a full antimicrobial susceptibility testing (AST). Two of such test systems allowing rapid diagnostics were assessed in this study: The Biofire FilmArray® and the Genmark ePlex®, both fully automated test system with a minimum of hands-on time. Overall 137 BSI episodes were included in our study and compared to conventional culture–based reference methods. The FilmArray® is using one catridge including a panel for the most common bacterial and fungal BSI pathogens as well as selected resistance markers. The ePlex® offers three different cartridges for detection of Gram-positives, Gram-negatives, and fungi resulting in a broader panel including also rare pathogens, putative contaminants, and more genetic resistance markers. The FilmArray® and ePlex® were evaluated for all 137 BSI episodes with FilmArray® detecting 119 and ePlex® detecting 128 of these. For targets on the respective panel of the system, the FilmArray® generated a sensitivity of 98.9% with 100% specificity on Gram-positive isolates. The ePlex® system generated a sensitivity of 94.7% and a specificity of 90.7% on Gram-positive isolates. In each case, the two systems performed with 100% sensitivity and specificity for the detection of Gram-negative specimens covered by each panel. In summary, both evaluated test systems showed a satisfying overall performance for fast pathogen identification and are beneficial tools for accelerating blood culture diagnostics of sepsis patients.

**Electronic supplementary material:**

The online version of this article (10.1007/s10096-020-03828-5) contains supplementary material, which is available to authorized users.

## Introduction

As bacteremia and sepsis are still leading causes of morbidity and mortality [1, 2], fast diagnosis of the causative organism and appropriate antimicrobial therapy are essential for rapid treatment decisions [3]. Worldwide, the incidence of sepsis is about 19 million per year [4] with a lethality of 20–30% [5, 6]. This emphasizes the need for rapid identification (ID) and detection of resistance genes. Conventional microbiological, agar-based methods used for blood culture diagnostics include culture-dependent identification by MALDI-TOF and AST by phenotypic methods. As these techniques are time-consuming, automated molecular ID methods with lower hands-on and turnaround time were developed allowing for identification and the genotypic detection of resistance genes [7–12]. Therefore, using quick molecular-based ID tests directly from positive blood culture bottles can help with providing an adequate therapy in a timely manner by de-escalating broad-spectrum antimicrobial therapy or escalating insufficient treatment, respectively [13–15].

To speed up the identification and AST of Gram-negative bacteria in positive blood cultures, the Accelerate Pheno® system (Accelerate® Diagnostics, USA) was evaluated for fast ID and AST compared to culture-based diagnostics [12]. The system is implemented in our routine laboratory accelerating reports for ID and AST especially for high-risk patients having BSI with Gram-negative bacteria. To establish a workflow for faster identification of all pathogens causing bloodstream infections, two different blood culture identification panels were compared in this study.

The BioFire FilmArray® Blood Culture Identification Panel (Biomerieux, Nürtingen, Germany) is a CE-marked multiplex PCR system, which allows identification of 24 targets at once including Gram-positives, Gram-negatives, and fungi, as well as three antibiotic resistance genes. DNA extraction, multiplex-PCR, and detection are fully automated with 2 min of hands-on time. A results report is generated approximately after 75 min [16].

The ePlex® system (Genmark Diagnostics, Carlsbad, USA) is endowed with three variable cartridges for pathogen identification. Deploying this test allows the detection of Gram-positives, Gram-negatives, and fungi. Besides the coverage of a large variety of organisms (55 bacterial/fungal targets) the test is designed to detect ten antibiotic resistance genes in addition. Nucleic acid extraction, amplification, and detection are completed in about 90 min using the microfluidic eSensor technology and require only 2 min of hands-on time.

The aim of the present prospective study was the comparative analysis of two molecular based Blood Culture Identification panels in comparison to conventional culture-dependent ID methods in terms of (i) correct detection and identification of the pathogen, (ii) correct prediction of resistance genes and genotypes, and (iii) suitability for implementation in a diagnostic routine workflow.

## Material and methods

### Study design

The study was performed from November 2017 to January 2018 at a tertiary university hospital in Southern Germany in accordance with the local ethics committee (no. 667/2014BO1 and 139/2016BO2).

The first positive blood culture of each patient with a positive initial Gram-stain (Gram-positive or Gram-negative bacteria as well as fungi) was included in the study until 100 cultures with Gram-positive isolates were reached. Samples having more than one obvious organism according to the Gram-stain were not included in the study.

**Fig. 1:**
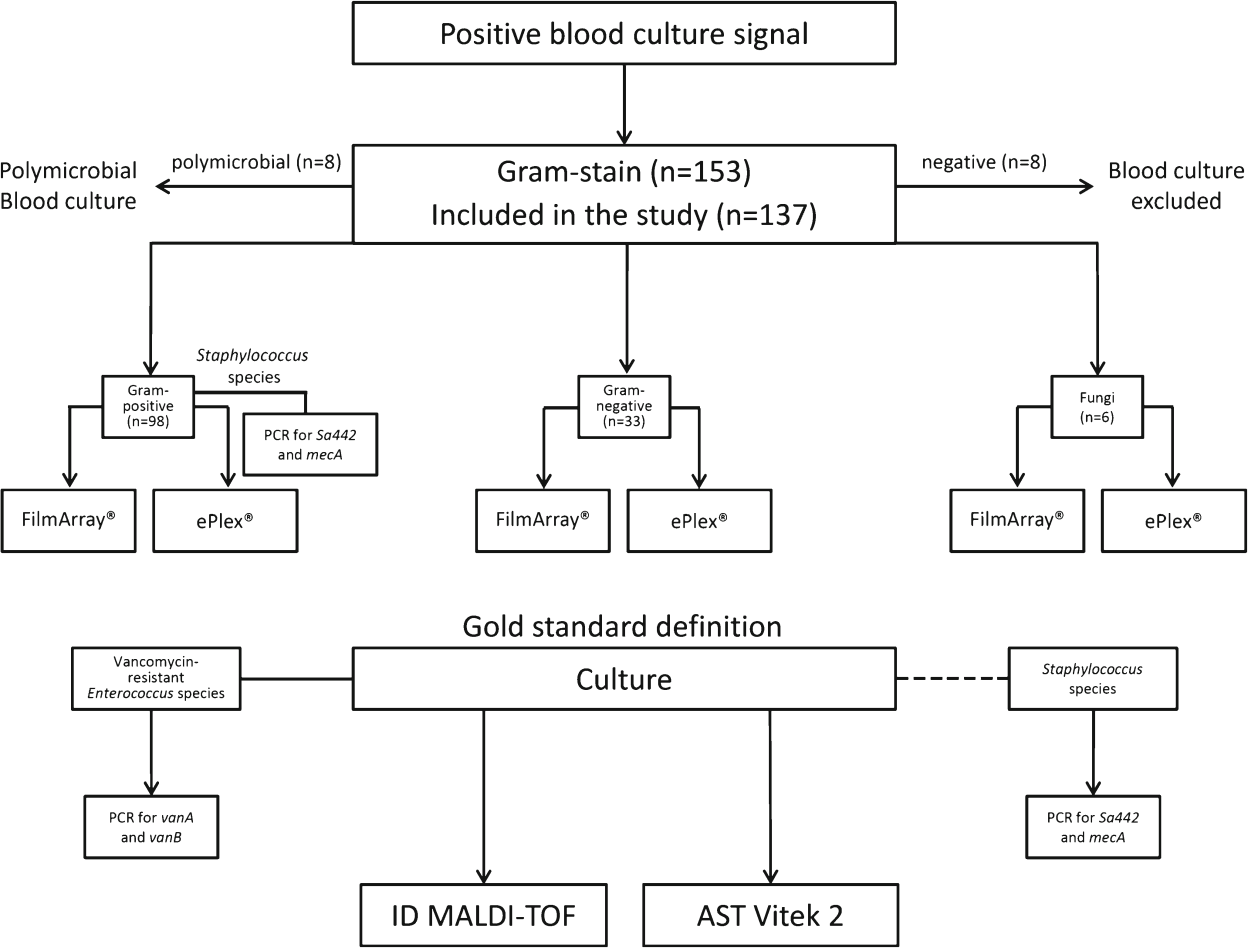
Flow chart of the study design. Each patient having a first positive blood culture signal (*n* = 153) with a positive initial Gram-stain (Gram-positive [*n* = 98] or Gram-negative bacteria [*n* = 33] as well as fungi [*n* = 6]) was included in the study (in total *n* = 137). Samples having more than one obvious organism according to the Gram-stain (*n* = 1) and Gram-stainings showing no organism (*n* = 8) were excluded from the study. Blood cultures positive for Gram-positive, Gram-negative organisms or fungi were analyzed with the FilmArray® and the ePlex® blood culture panels. On weekdays, PCR for *Staphylococcus aureus* and the *mecA* gene was performed directly from positive blood culture bottles, based on the Gram-stain result indicating the presence of *staphylococci*. Results obtained from the two evaluated test systems were compared to culture- (MALDI-TOF identification and Vitek-AST) or molecular-based (in-house PCR for *Sa442* and *mecA* or *vanA/vanB* respectively) reference method

Positive blood cultures with Gram-positive (*n* = 98) or Gram-negative bacteria (*n* = 33) as well as fungi (*n* = 6) were analyzed by two different blood culture identification instruments: FilmArray® Blood Culture Identification panel (BioFire Diagnostics, Salt Lake City, UT, USA) and ePlex® Research Use Only (RUO) Blood Culture Identification panels (Genmark Diagnostics, Carlsbad, CA, USA). A schematic overview of the study design is shown in Fig. 1.

### Conventional diagnostics

Laboratory operation hours are weekdays from 7:30 AM to 5:30 PM and weekends from 7:30 AM to 4 PM. Blood cultures are processed every morning starting at 7:30 AM and during the operating hours as soon as they are flagged positive.

Incubation of Blood culture bottles was performed in the Bactec FX blood culture instrument (BD Diagnostic Systems, Franklin Lakes, USA). The routine diagnostic workflow included subcultures of positive blood culture bottles on CNA- (Biomerieux, Nürtingen, Germany) and Brain Heart agar (Oxoid, Munich, Germany) in addition to Gram-stain. Identification to the species level was achieved by MALDI-TOF mass spectrometry (Microflex LT, Bruker Daltonics, Germany) and supplemented, if necessary, by the VITEK® 2 identification system (bioMérieux SA, France).

The appropriate testing card of VITEK® 2 (bioMérieux, SA, France) was used for Antimicrobial Susceptibility Testing (AST). All bacterial isolates were stored at − 80 °C for further analysis. The guidelines set of the European Committee on Antimicrobial Susceptibility Testing (EUCAST) (http://www.eucast.org/fileadmin/src/media/PDFs/EUCAST_files/Breakpoint_tables/v_8.1_Breakpoint_Tables.pdf) were consulted for interpretation of susceptibility results.

If necessary, resistance genes *bla*_VIM_, *bla*_IMP_, *bla*_KPC_, *bla*_OXA-48_, and *bla*_NDM_ for carbapenemases were confirmed by molecular diagnostics (Light Mix Modular Realtime-PCR, Roche, Mannheim). In house-PCR for *mecA* [17] was performed on a T3 thermal cycler (Biometra, Göttingen) and analyzed by agarose gel electrophoresis directly from positive blood culture bottles during laboratory operating hours, but not during weekends. For these cases, the *mecA*-PCR was repeated from culture isolates. *Enterococcus* species were tested for the presence of vancomycin resistance genes *vanA* and *vanB* by in-house PCRs [18] using T3 thermal cycler (Biometra, Göttingen) followed by gel electrophoresis. Resistance of selected *Enterobacterales* against 3rd generation cephalosporins was further analyzed phenotypically for the presence of extended-spectrum beta lactamases (*bla*_CTX-M_) using ESBL detection disk set (Mast Diagnostica GmbH, Reinfeld, Germany). 16S rRNA-PCR directly from positive blood culture bottles [17] using T3 thermal cycler (Biometra, Göttingen) was performed in case of lacking cultural growth.

Fungi were cultivated on yeast agar supplemented with gentamicin (in-house preparation). Single colonies were identified with MALDI-TOF mass spectrometry (Microflex LT, Bruker Daltonics, Germany) and BBL CHROMagar for Candida (BD Diagnostic Systems, Franklin Lakes, USA). Resistance profiles for fungi were not assessed for the study.

### Biofire FilmArray® blood culture identification panel

The FilmArray® instrument was run according to manufacturer’s instructions. In brief, the reagents in the pouch were adjusted with approximately 300 μl rehydration solution. Next, 100 μl medium from the positive blood culture bottle was mixed with sample buffer and loaded into the pouch, which requires only 2 min hands-on time. Once inserted in the FilmArray® instrument, nucleic acid extraction was performed followed by nested multiplex PCR giving results in about 1 h. The BioFire FilmArray® is using one reagent pouch for identification of Gram-positives, Gram-negatives, and fungi. The organisms covered by the assay are shown in Table S1. The software of the FilmArray® generates an automated report. One system was available during the study period to analyze one sample at a time.

### Genmark ePlex® BC panels

The GenMark ePlex® system is using the eSensor technology deploying competitive DNA hybridization followed by electrochemical detection. Three different blood culture identification panels are available: one cartridge each for the identification of Gram-positives (BCID-GP), Gram-negatives (BCID-GN), and fungi (BCID-FP). The decision of which cartridge to use is based on the Gram-stain result. To run the assay in accordance with the manufacturer specifications, 50 μl of positive blood culture are transferred into the sample port of the respective cartridge followed by loading into the system. One tower with six test bays was available during the study period, offering the possibility to analyze six blood culture samples simultaneously. Result reports were generated automatically after about one and half hours from starting the test. Organisms included in the panel are illustrated in Table S1. The cartridges for Gram-negative and Gram-positive bacteria include a PAN target, that detects the presence of Gram-positives organisms in the Gram-negative (BCID-GN) cartridge and vice versa helping to identify polymicrobial mixed BSI that may have been missed by Gram-staining initially. In addition, a PAN-target for *Candida* is included on the bacterial detection cartridges.

### Data analysis

Identification of blood culture isolates by MALDI-TOF was defined as reference method. In case of lacking growth, 16S rRNA-PCR was performed directly out of positive blood culture bottles. Dependent on the fast ID system tested, some species are only identified to the genus level. Exact genus ID—as identified by the reference method—was rated as correctly identified in case a fast ID system did not provide species level identification. If one of the two systems produced an invalid or failed run, it was repeated with a new identification cartridge. Due to operating hours of the laboratory during the study period, it was not possible to repeat every invalid or failed run generated by one of the two test systems. Therefore two samples (*n* = 2) were excluded from the study.

Detection of genotypic resistance markers by the test systems were compared to the results of in-house PCRs in relation to the phenotypic resistance obtained by Vitek 2 or agar diffusion (ESBL screening) respectively.

An identification was called ‘true positive’ for any organism covered by the FilmArray® or the ePlex® panel respectively and identified by the reference method as well as by FilmArray® or the ePlex®. ‘True negative’ was defined as any species not detected by the panels of the two systems and different from the reference method result. Organisms are called ‘false positive’ if identified by the FilmArray® or ePlex® system and not by the reference method. Any organism missed or misidentified by the two ID systems, but detected by the reference method, were marked as “false negative.”

The calculated specificity and the number of true negatives refer to positive blood cultures, which were negative for the respective target organism.

## Results and discussion

During the study period, 153 positive blood cultures gave a positive signal and were assessed for inclusion in our analysis. Overall, eight positive blood cultures were excluded from the study, due to a negative initial Gram-stain result. In total, 137 positive blood cultures were included in our study (Fig. 1). Among those 98 episodes were monomicrobial BSI with Gram-positive organisms, 33 were monomicrobial with Gram-negative organisms and 6 episodes were caused by yeast strains (Tab.1). In addition, in eight samples, more than one organism was detected by culture. One example was not included in the study as the presence of multiple organisms was already suspected by the Gram stain, the results of the remaining seven samples are summarized in Table S2.

**Table 1:** Identification results for bacteria and yeast (monomicrobial BSI)

	No. of true/false positives no. of true/false negatives		No. of true/false positives no. of true/false negatives		
Species (*n* = reference method ID)	FilmArray®	ePlex®	FilmArray®	ePlex®	Comments
ID of Gram-positive organisms					
*S. aureus* (*n* = 18)	18/0	18/0	119/0	119/0	
*S. epidermidis* (*n* = 35)	35/0^a^	35/2	102/0	100/0	FilmArray® ID only on *Staphylococcus* spp. level, false ePlex® ID of *S. hominis* as *S. epidermidis*, false ePlex® ID of *S. warneri* as *S. epidermidis*
*S. haemolyticus* (*n* = 8)	8/0^a^	7/0	129/0	129/1	FilmArray® ID only on *Staphylococcus species* level, no ePlex® ID for one *S. haemolyticus* isolate
*S. hominis* (*n* = 6)	6/0^a^	5/0	131/0	131/1	FilmArray® and ePlex® ID only on *Staphylococcus* spp. level, false ePlex® ID of *S. hominis* as *S. epidermidis*
*S. warneri* (*n* = 2)	1/0^a^	1/0	135/1	135/1	FilmArray® and ePlex® ID only on *Staphylococcus* spp. level, no FilmArray® ID for 1/2 isolates, false ePlex® ID of one *S. warneri* isolate as *S. epidermidis*
*S. capitis* (*n* = 2)	2/0^a^	2/0	135/0	135/0	FilmArray® and ePlex® ID only on *Staphylococcus* spp. level
*E. faecium* (*n* = 4)	4/0^a^	4/2	133/0	131/0	FilmArray® ID only on *Enterococcus* spp. level, false ePlex® ID for two *E. faecalis* isolates as *E. faecium*
*E. faecalis* (*n* = 5)	5/0^a^	3/0	132/0	132/2	FilmArray® ID only on *Enterococcus* spp. level, false ePlex® ID for two *E. faecalis* isolates as *E. faecium*
*S. pneumoniae* (*n* = 1)	1/0	1/0	136/0	136/0	
*S. gallolyticus* (*n* = 2)	2/0	2/0	135/0	135/0	FilmArray® and ePlex® ID only on *Streptococcus* spp. level
*S. mitis group* (*n* = 1)	1/0	1/0	136/0	136/0	FilmArray® and ePlex® ID only on *Streptococcus* spp. level
*S. oralis* (*n* = 1)	1/0	1/0	136/0	136/0	FilmArray® and ePlex® ID only on *Streptococcus* spp. level
*S. anginosus* (*n* = 1)	1/0	1/0	136/0	136/0	FilmArray® ID only on *Streptococcus* spp. level
*Streptococcus sp.* (group C) (n = 1)	1/0	1/0	136/0	136/0	FilmArray® and ePlex® ID only on *Streptococcus* spp. level
*C. acnes* (*n* = 5)	n/a	5/0	n/a	132/0	Not included in the FilmArray® panel
*A. meyeri* (*n* = 1)	n/a	n/a	n/a	n/a	Not included in the FilmArray® and ePlex® panel
*Actinomyces sp.* (*n* = 1)	n/a	n/a	n/a	n/a	Not included in the FilmArray® and ePlex® panel
*P. faecalis* (*n* = 1)	n/a	n/a	n/a	n/a	Not included in the FilmArray® and ePlex® panel
*Lactobacillus sp.* (*n* = 1)	n/a	1/0	n/a	136/0	Not included in the FilmArray® panel, ePlex® ID only on *Lactobacillus* spp. level
*Corynebacterium sp.* (*n* = 1)	n/a	1/0	n/a	136/0	Not included in the FilmArray® panel
*A. parvulum* (*n* = 1)	n/a	n/a	n/a	n/a	Not included in the FilmArray® and ePlex® panel
Total number of organisms (*n* = 98)	86/0	89/4	50/1	39/5	
ID of Gram-negative organisms					
*E. coli* (*n* = 15)	15/0	15/0	122/0	122/0	
*K. pneumoniae*/*variicola* (*n* = 5)	5/0	5/0	132/0	132/0	Two *K. variicola* identified as *K. pneumoniae* by FilmArray® and ePlex®
*K. oxytoca* (*n* = 1)	1/0	1/0	136/0	136/0	
*S. marcescens* (*n* = 2)	2/0	2/0	135/0	135/0	
*E. cloacae Komplex* (*n* = 1)	1/0	1/0	136/0	136/0	
*E. ludwigii* (*n* = 1)	1/0	1/0	136/0	136/0	FilmArray® and ePlex® ID as *E. cloacae* complex
*P. aeruginosa* (*n* = 1)	1/0	1/0	136/0	136/0	
*H. influenzae* (*n* = 1)	1/0	1/0	136/0	136/0	
*M. liquefaciens* (*n* = 1)	n/a	n/a	n/a	n/a	Not included in the FilmArray® and ePlex®panel
*N. polysaccharea* (*n* = 1)	n/a	n/a	n/a	n/a	Not included in the FilmArray® and ePlex® panel
*C. canimorsus* (*n* = 1)^a^	n/a	n/a	n/a	n/a	Not included in the FilmArray® and ePlex®panel; ^a^only identified by 16S-rRNA-PCR
*B. fragilis (n = 2)*	n/a	2/0	n/a	135/0	Not included in the FilmArray® panel
*B. vulgatus/dorei* (*n* = 1)	n/a	n/a	n/a	n/a	Not included in the FilmArray® and ePlex® panel
Total number of organisms (*n* = 33)	27/0	29/0	110/0	108/0	
ID of yeasts					
*Candida albicans* (*n* = 2)	2/0	2/0	135/0	135/0	
*Candida glabrata* (*n* = 2)	2/0	2/0	135/0	135/0	
*Candida parapsilosis* (*n* = 1)	1/0	1/0	136/0	136/0	
*Candida tropicalis* (*n* = 1)	1/0	1/0	136/0	136/0	
Total number of organisms (*n* = 6)	6	6	131/0	131/0	

^a^Coagulase-negative *Staphylococcus* spp. as well as *Enterococcus* spp. were detected on genus level only by the FilmArray®

### Identification of Gram-positive BSI pathogens

In summary 21 different Gram-positive organisms (98 isolates in total) were determined by routine-diagnostic methods. Identification by MALDI-TOF was compared to identification results created by the BioFire FilmArray® and the GenMark ePlex® system.

Correct identification by the FilmArray® was achieved for 86/98 (87.8%) isolates on genus and species level, as defined in the system panel for the respective target organism. The system scored 12 samples negative (12.2%). These 11 samples contained organisms that are not included in the system panel, namely *C. acnes* (*n* = 5*), Actinomyces meyeri* (*n* = 1), *Actinomyces species* (n = 1), *P. faecalis* (n = 1), *Lactobacillus species* (n = 1), *Corynebacterium species* (n = 1) and *Atopobium parvulum* (n = 1). One *Staphylococcus warneri* isolate was not detected as *Staphyloccus species* by the system. ID failure of *S. warneri* has been reported before and was explained by a reduced sensitivity of the FilmArray® system for some CoNS including *S. warneri* [19], which is also mentioned in the package insert of the panel. The FilmArray® therefore identified 86/87 (98.9%) correctly taking only the organisms included in the panel into account. The results are shown in detail in Table 1. For the Gram-positives, the FilmArray® showed a sensitivity of 98.9% and a 100% specificity (Table 4) for organisms covered by the panel.

In contrast to the FilmArray® system, the ePlex® system aims at identifying enterococci and coagulase negative staphylococci to the species level. In total identification to the species level was achieved in 89/98 (90.8%) of the Gram-positive bacterial isolates. Two *Staphyloccus* species were misidentified by the system: *S. hominis* as well as *S. warneri* were identified as *S. epidermidis*. A larger sample size is required to assess if this assay is more prone to misidentification of CoNS. Furthermore, it is difficult to rule-out that one or more of these specimens contained multiple *Staphylococu*s species. In addition, as all these three CoNS species belong to the *S. epidermidis* group as part of the normal skin flora with similar pathogenicity [20] it is not mandatory to identify these CoNS on the species level. Worth mentioning is the inclusion of *S. lugdunensis* in the Genmark ePlex® panel as it is a common pathogen with species specific virulence factors [21] associated with aggressive causes of infective endocarditis [22–24].

In addition, two *E. faecalis* isolates were misidentified as *E. faecium.* The correct identity of these two *E. faecalis* isolates was verified by MALDI-TOF and confirmed by next generation sequencing (NGS) of the whole genome on an Illumina Nextseq platform followed by calculating the average nucleotide identity (ANI) [25]. The ePlex® cartridges used in this study were for Research Use Only and according to the package insert of the test assay in a subsequent clinical study evaluating the performance of the CE-IVD and U.S. FDA cleared assays, the ePlex® demonstrated high sensitivity and specificity for identification of *Enterococcus faecalis*. *Enterococcus* species cause approximately 10% of nosocomial blood stream infections [26] caused by a variety of different entries of the pathogen [27]. Treatment of *Enterococcus* related BSI is strongly dependent on correct species identification. Whereas *E. faecalis* is mostly susceptible to the small-spectrum antibiotic ampicillin, *E. faecium* is resistant [28].

Classifying CoNS and enterococci only to the genus level, correct identification could be achieved for 93/98 (94.9%) of the Gram-positive bacterial isolates. Based on the broader panel *C. acnes* (*n* = 5), *Lactobacillus species* (*n* = 1) and *Corynebacterium species* (*n* = 1) were detected in contrast to the FilmArray® system. Four bacterial isolates were not identified as they were not part of the panel: *Actinomyces meyeri* (*n* = 1), *Actinomyces species* (*n* = 1), *P. faecalis* (*n* = 1) and *Atopobium parvulum* (*n* = 1). Moreover one *S. haemolyticus* strain was not detected by the system. Regarding only organisms covered by the panel, the ePlex® correctly identified 93/94 (98.9%) strains to genus level and 89/94 (94.7%) to the species level. For two blood cultures, the ePlex® detected a second Gram-positive organism (*Micrococcus* in addition to *S. aureus* and accordingly *Staphylococcus spp.* in addition to *E. faecium*). None of these additional organisms could be confirmed by culture (Table 1). The ePlex® panel allows in contrast to the FilmArray® identification of potential contaminants namely *Corynebacterium* spp., *Micrococcus* spp., and *C. acnes* usually resulting in no need for treatment unless there is direct evidence for infection by the organism. Fast identification of such contaminants can help in quick de-escalation of the ongoing therapy and prevent redundant administration of anti-infectious therapies [29–31].

The ePlex® system showed a sensitivity of 94.7% and a specificity of 90.7% for Gram-positive pathogens included in the panel (Table 4).

Percentages of detected organisms covered by the respective blood culture identification panel as well as for all microorganism detected by the reference method are shown in Table 2.

**Table 2:** Resistance genes identified for Gram-positive bacteria by FilmArray® and ePlex®

Genotypic detection of methicillin resistance (*mecA*/*mecC* )						
*Staphylococcus* species (total *n*=66)	*mecA* in house PCR	*mecC* in house PCR	*mecA* FilmArray®	*mecA* ePlex®	*mecC* ePlex®	comment
*S. aureus* (*n*=18)	negative (*n*=18)^a^	n/a	negative (*n*=18)	negative (*n*=18)	negative (*n*=18)	
Coagulase-negative *Staphylococcus spp.* (*n*=48)	negative (*n*=15)^b^	n/a	negative (*n*=16)	negative (*n*=15)	negative (*n*=48)	False-negative mecA result by FilmArray® (*n*=1)
	positive (*n*=33)^c^		positive (*n*=31)	positive (*n*=33)		No target detected by FilmArray® (*n*=1)
Genotypic detection of vancomycin resistance *(vanA*/*vanB*)						
*Enterococcus* species (total *n*=9)	*vanA*/*vanB* in-house PCR	*vanA*/*vanB* FilmArray®	*vanA*/*vanB* ePlex®		comment	
*Enterococcus* vancomycin sensitive (*n*= 8)	negative (*n*=8)	negative (*n*=8)	negative (*n*=8)		*E. faecalis* (*n*=5), *E. faecium* (*n*=3)	
*Enterococcus* vancomycin resistant (*n*= 1)	positive *van* B (*n*=1)	positive *vanA* /*vanB* (*n*=1)	positive *vanB* (*n*=1)		*vanB* positive *E. faecium* isolate	

^a^tested from blood culture (*n*=13), tested from culture (*n*=5)

^b^tested from blood culture (*n*=11), tested from culture (*n*=4)

^c^tested from blood culture (*n*=18), tested from culture (*n*=15)

### Identification of Gram-negative BSI pathogens

We further assessed identification of Gram-negative organisms for the BioFire FilmArray® and the Genmark ePlex® system. Routine-diagnostic analysis provided evidence for 14 different species within 33 samples in total. Due to the lack of bacterial growth, one sample with *Capnocytophaga canimorsus* was not verified by MALDI-TOF, but by 16S rRNA PCR out of the positive blood culture bottle. Two *Klebsiella variicola* isolates were reported as *Klebsiella pneumoniae* by the FilmArray® and GenMark ePlex®, but were not assessed as misidentification, as MALDI-TOF based differentiation of these two species has only recently become available [32]. *Klebsiella variicola* was described as a new species genetically isolated from *K. pneumoniae* and can be phenotypically distinguished by the inability of adonitol fermentation. The pathogenicity potential and analysis of virulence factors of *K. variicola* are still under investigation and high-risk antibiotic resistance genes are already described for *K. variicola* [33–35]. Thus, identification of *K. variicola* on species level and dissociation from *K. pneumoniae* could be of relevance for a future panel update.

In contrast to the FilmArray® panel, the ePlex® system can identify the *Bacteroides fragilis*, which is of clinical importance since the *B. fragilis* group belongs to the most prevalent anaerobic pathogens causing BSI [36].

In total, the FilmArray® identified 27/33 isolates (81.8%). Six samples included organisms outside the panel (*Moraxella liquefaciens* (*n* = 1), *Neisseria polysaccharea* (*n* = 1), *Capnocytophaga canimorsus* (*n* = 1), *B. fragilis* (*n* = 2) and *B. vulgatus/dorei* (*n* = 1)). This resulted in a correct identification by the FilmArray® instrument for all 27/27 (100%) organisms included in the panel.

The ePlex® system identified 29/33 isolates correctly (87.9%), whereas *M. liquefaciens* (*n* = 1), *N. polysaccharea* (*n* = 1), *C. canimorsus* (*n* = 1) and *B. vulgatus/dorei* (*n* = 1) were not included in the panel. Thus, the remaining 29/29 specimens (100%) were correctly identified.

### Identification of yeast strains as BSI pathogens

During the study period, six BSI episodes with different yeast strains occurred. Both Biofire FilmArray® and GenMark ePlex® identified all 6/6 strains (100%) correctly, namely *C. albicans* (*n* = 2), *C. glabrata* (*n* = 2), *C. parapsilosis* (*n* = 1) and *C. tropicalis* (*n* = 1), which were all confirmed by MALDI-TOF analysis.

### Detection of polymicrobial BSI pathogens

In total, for seven polymicrobial BSI episode samples, both test systems were performed (summarized in Table S2). The FilmArray® and ePlex® detected all pathogens covered by the panels at least on genus level with only one exception: the FilmArray® missed detecting a *Streptococcus constellatus* isolate although covered by the panel, which was found in addition to *Proteus mirabilis* in one blood culture bottle. In the initial Gram-stain, only Gram-negative rods were observed. Furthermore, culture-based diagnostic showed only a few colonies of *Streptococcus* on CNA agar plates, indicating a low burden of Gram-positives in this blood culture.

For the ePlex® the cartridge for Gram-negatives was used for the analysis of that blood culture resulting in the detection of the Gram-positive PAN target indicating the precence of the *Streptococcus constellatus* isolate. The Gram-positive PAN target was also detected in a polymicrobial BSI episode containing *S. epidermidis* in addition to *K. pneumoniae*.

In another polymicrobial blood culture, *Fusobacterium nucleatum* and *Dialister pneumosintes* were identified by culture. In the initial Gram-stain, the Gram-negative rods appeared Gram-positive, whereupon the ePlex® cartridge for Gram-positives was used. Both species are not covered by the FilmArray® panel and were not detected accordingly. However, no PAN target for Gram-negatives was detected by the ePlex®.

### Detection of genotypic resistance markers

In total, 71 *Staphylococcus species* were identified in the study. Five *Staphylococcus* isolates could not be verified for the presence of *mecA* by in-house PCR as the isolates were not stored by the reason of routine laboratory workflow. Nevertheless, the susceptibility results of these five *Staphylococcus* isolates correlate with the detection results for *mecA* generated by the FilmArray® and the ePlex®. For the residual 66 *Staphylococcus* isolates in-house *mecA*-PCR results were available. All *S. aureus* isolates (*n* = 18) were methicillin-susceptible and negative for *mecA* as reported by the FilmArray®, the ePlex® and the in-house PCR (Table 3). Moreover, no *mecC* was detected by the ePlex® system. In the 48 BSI episodes with CoNS, 15 isolates were methicillin-susceptible and 33 methicillin-resistant according to VITEK 2 results and *mecA* in-house PCR. This is in accordance with the *mecA*-PCR results of the ePlex® system. The FilmArray® produced one false-negative *mecA* result for an oxacillin-resistant *S. epidermidis* isolate positive for *mecA* in the in-house PCR. For the *S. warneri* isolate not identified by the system no PCR result was produced. Other, recently evaluated option for accelerated AST results include the culture-based EUCAST rapid antimicrobial susceptibility testing (RAST) allowing interpreting inhibition zones after 4, 6, or 8 h of growth (The European Committee on Antimicrobial Susceptibility Testing. Zone diameter breakpoints for rapid antimicrobial susceptibility testing (RAST) directly from blood culture bottles. Version 1.0, 2018. http://www.eucast.org.).

**Table 3:** Resistance genes identified for Gram-positive bacteria by FilmArray® and ePlex®

Genotypic detection of methicillin resistance (*mecA*/*mecC*)						
*Staphylococcus* species (total *n* = 66)	*mecA* in house PCR	*mecC* in house PCR	*mecA* FilmArray®	*mecA* ePlex®	*mecC* ePlex®	Comment
*S. aureus* (*n* = 18)	Negative (*n* = 18)^a^	n/a	Negative (*n* = 18)	Negative (*n* = 18)	Negative (*n* = 18)	
Coagulase-negative *Staphylococcus* spp. (*n* = 48)	Negative (*n* = 15)^b^	n/a	Negative (*n* = 16)	Negative (*n* = 15)	Negative (*n* = 48)	False-negative mecA result by FilmArray® (*n* = 1)
	Positive (*n* = 33)^c^		Positive (*n* = 31)	Positive (*n* = 33)		No target detected by FilmArray® (*n* = 1)
Genotypic detection of vancomycin resistance *(vanA*/*vanB*)						
*Enterococcus* species (total *n* = 9)	*vanA*/*vanB* in-house PCR	*vanA*/*vanB* FilmArray®	*vanA*/*vanB* ePlex®	Comment		
*Enterococcus* vancomycin sensitive (*n* = 8)		negative (*n* = 8)	negative (*n* = 8)	negative (*n* = 8)	*E. faecalis* (*n* = 5), *E. faecium* (*n* = 3)	
*Enterococcus* vancomycin resistant (*n* = 1)		positive *van* B (*n* = 1)	positive *vanA* /*vanB* (*n* = 1)	positive *vanB* (*n* = 1)	*vanB* positive *E. faecium* isolate	

^a^Tested from blood culture (*n* = 13), tested from culture (*n* = 5)

^b^Tested from blood culture (*n* = 11), tested from culture (*n* = 4)

^c^Tested from blood culture (*n* = 18), tested from culture (*n* = 15)

During the study period, nine *Enterococcus* species (*E. faecalis* [*n* = 5] and *E. faecium* [*n* = 4]) were isolated. Eight isolates were negatively tested for the presence of *vanA* and *vanB* by routine-diagnostic in-house PCR as well as by the FilmArray® and ePlex® system. One vancomycin resistant isolate (positive for *vanB*) was identified by FilmArray® and ePlex® (Tab. 3). None of the 33 isolated Gram-negative microorganisms were carbapenem-resistant, being in line with negative PCR results from the evaluated systems and reflecting the low prevalence of BSI with carbapenem-resistant bacteria in our hospital [37]. However, due to increasing bacterial resistance, rapid detection of genes encoding carbapenemases is a desirable characteristic of molecular-based blood culture identification panels. In this regard the carbapenemase resistance genes covered by the ePlex® panel for Gram-negatives can be highlighted as the most common genes [38]. The ePlex® detected the presence of *bla*_CTX-M_ for three study isolates (*E. coli* (*n* = 2) and *K. pneumoniae* (*n* = 1)), phenotypically confirmed by the presence of an ESBL phenotype in the cultured strains.

A summary of the overall performance of the two test systems is illustrated in Table 4. Concerning the detection of Gram-positive bacteria included in the panels of the FilmArray® and the ePlex®, the best performance with slightly higher sensitivity, specificity, PPV and NPV values was shown by the FilmArray® (Table 4). However, due to the broader identification panel of the ePlex®, putative contaminants or frequent anaerobes can be identified, resulting in on overall higher number of identified Gram-positive organisms (93/98) as compared to the FilmArray® with identification of 86 of 93 specimens. Regarding the identification of Gram-negative organisms and yeast strains, no major differences were found between the two systems.

**Table 4:** Overview of sensitivity, specificity, positive predictive values (PPV), and negative predictive values (NPV) for in-panel organisms only

	FilmArray®	ePlex®
Sensitivity (%)		
Gram-positives	98.9	94.7
Gram-negatives	100	100
Specificity (%)		
Gram-positives	100	90.7
Gram-negatives	100	100
Positive predictive value (PPV) (%)		
Gram-positives	100	95.7
Gram-negatives	100	100
Negative predictive value (NPV) (%)		
Gram-positives	98	88.6
Gram-negatives	100	100

In conclusion and in view of our study aims, both blood culture identification systems showed good results for fast pathogen recognition directly from positive blood cultures as well as for resistance gene detection. The broad coverage three panel approach of the ePlex® system implicate a drawback as it necessitates a Gram-staining prior to decide which cartridge to use for identification. However, the intended use of the other system also requires a Gram-stain as part of their approved instructions for use. Both systems require only short hands-on time, can easily be implemented in a routine microbiological diagnostic workflow and cause similar costs.

The Accelerate Pheno® system, which is already implemented for high risk patients in our blood culture workflow [12], allows accelerating reports for ID and especially full AST for hard to predict resistance profiles for Gram-negatives. The panels of the FilmArray® and ePlex® systems with their ability for detecting common antimicrobial resistance genes are suitable for fast identification and rough genotypic resistance characterization of Gram-positive organisms, specifically for *Staphylococcus* species demarcating *S. aureus* and MRSAs and *Enterococcus* species defining VREs. Specific performance characteristics of the assays, cost-benefit ratio, laboratory operating hours, manning and state of knowledge of the laboratory personnel, patient population as well as effect on patient care from individual identification panels affect the advantages of rapid PCR-based blood culture diagnostics implicating the need of a considered selection and implementation of a rapid molecular ID system [39].

## Electronic supplementary material

ESM 1 (DOCX 32 kb)
