# Supplementary material for: Evaluation of two rapid molecular test systems to establish an algorithm for fast identification of bacterial pathogens from positive blood cultures
Source: Eur J Clin Microbiol Infect Dis. 2020 Feb 4;39(6):1147–57. doi: 10.1007/s10096-020-03828-5 (PMC7225181; doi:10.1007/s10096-020-03828-5)
Supplement: Supplementary file 1 — (DOCX 32 kb) [file 10096_2020_3828_MOESM1_ESM.docx]

**Supplementary table 1:** Targets included in each panel of the two identification systems (A: BioFire FilmArray®, B: Genmark ePlex®)

**Supplementary table 2:** Identification results for bacteria (polymicrobial BSI)
